# Partial Desalination of Saline Groundwater: Comparison of Nanofiltration, Reverse Osmosis and Membrane Capacitive Deionisation

**DOI:** 10.3390/membranes11020126

**Published:** 2021-02-12

**Authors:** Hanna Rosentreter, Marc Walther, André Lerch

**Affiliations:** 1 Chair of Process Engineering in Hydro Systems, Institute of Urban and Industrial Water Management, Faculty of Environmental Sciences, Technische Universität Dresden, 01062 Dresden, Germany; andre.lerch@tu-dresden.de; 2 Chair of Forest Biometrics and Forest Systems Analysis, Department of Forest Sciences, Faculty of Environmental Sciences, Technische Universität Dresden, 01062 Dresden, Germany; marc.walther@tu-dresden.de

**Keywords:** brackish water, design software, energy consumption, flexibility, managed aquifer recharge, mixed ion solution

## Abstract

Saline groundwater (SGW) is an alternative water resource. However, the concentration of sodium, chloride, sulphate, and nitrate in SGW usually exceeds the recommended guideline values for drinking water and irrigation. In this study, the partial desalination performance of three different concentrated SGWs were examined by pressure-driven membrane desalination technologies: nanofiltration (NF), brackish water reverse osmosis (BWRO), and seawater reverse osmosis (SWRO); in addition to one electrochemical-driven desalination technology: membrane capacitive deionisation (MCDI). The desalination performance was evaluated using the specific energy consumption (SEC) and water recovery, determined by experiments and simulations. The experimental results of this study show that the SEC for the desalination of SGW with a total dissolved solid (TDS) concentration of 1 g/L by MCDI and NF is similar and ranges between 0.2–0.4 kWh/m^3^ achieving a water recovery value of 35–70%. The lowest SECs for the desalination of SGW with a TDS concentration ≥2 g/L were determined by the use of BWRO and SWRO with 0.4–2.9 kWh/m^3^ for a water recovery of 40–66%. Even though the MCDI technique cannot compete with pressure-driven membrane desalination technologies at higher raw water salinities, this technology shows a high selectivity for nitrate and a high potential for flexible desalination applications.

## 1. Introduction

Due to a continuously growing population, economic development, and changing consumption patterns, potable water consumption has increased globally. At the same time, the quality of groundwater and surface water has decreased, causing a reduction in the levels of terrestrial water storage and water scarcity in regions such as the Middle East, India, Australia, and Africa [1,2,3,4]. Salinization can be both natural and anthropogenically induced and is caused by the long-term degradation of water quality in surface and groundwater resources [5]. In particular, coastal aquifers in semiarid and arid areas can be affected by saltwater intrusion [6,7,8,9]. Based on the mass concentration of chloride ( ${\mathrm{Cl}}^{-}$ ) and total dissolved solids (TDS), saline groundwater (SGW) is classified as either slightly SGW, moderately SGW or highly SGW [10,11], as listed in Table 1. Due to high concentrations of salt ions (e.g., sodium ( ${\mathrm{Na}}^{+}$ ), ${\mathrm{Cl}}^{-}$ , sulphate ( ${\mathrm{SO}}_{4}^{2-}$ ), and nitrate ( ${\mathrm{NO}}_{3}^{-}$ ))—exceeding the corresponding drinking water and irrigation guidelines—SGW is generally not suitable for either potable use or agricultural use [12,13]. However, compared to seawater, the ion concentration of SGW is often lower, making SGW a better option for energy efficient desalination [14,15].

**Figure 1:**
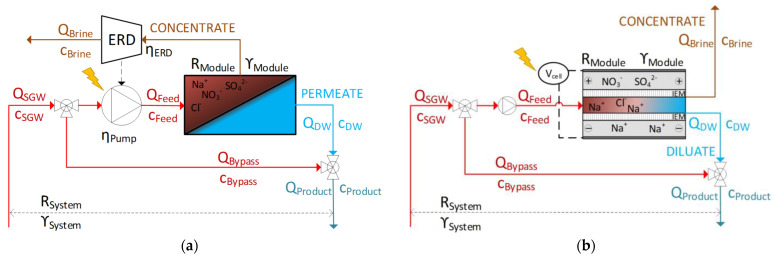
Process control diagram: (**a**) pressure-driven membrane desalination (e.g., reverse osmosis (RO) or nanofiltration (NF)); (**b**) membrane capacitive deionisation (MCDI) ( $Q_{SGW}$ : flow rate of saline groundwater (m^3^/h), $Q_{Feed}$ : flow rate of feedwater (m^3^/h), $\;Q_{DW}$ : flow rate of partial desalinated water (m^3^/h), $\;Q_{Bypass}$ : flow rate of the bypass (m^3^/h), $\;Q_{Product}$ : flow rate of the product (m^3^/h), $\;Q_{Brine}$ : flow rate of brine (m^3^/h),  c : molar salt concentration (mol/L), $\;R_{Module}$ : salt rejection of module (%), $\gamma_{Module}$ : water recovery of module (%), $R_{System}$ : salt rejection of system (%), $\gamma_{System}$ : water recovery of system (%), $\eta_{ERD}$ : efficiency of energy recovery device (%), $\eta_{Pump}$ : efficiency of pump (%), $V_{cell}$ : cell voltage (V), IEM: ion exchange membrane).

Desalination is a separation process used to reduce the concentration of TDS in saline water. Desalination technologies can be classified by the separation procedure. Either the pure water is removed from the feedwater as is the case in distillation and pressure-driven membrane desalination, or the salts are removed from the feedwater as is done in electrodialysis and capacitive deionisation [16]. At present, reverse osmosis (RO), which is a pressure-driven membrane desalination technique, is the most common desalination process for seawater and brackish water, due to its low energy consumption [16,17]. RO uses hydrostatic pressure greater than the osmotic pressure of the saline solution to drive the liquid through a membrane against the natural direction of osmosis producing a permeate stream on the effluent site and a concentrate stream (brine) on the influent site of the membrane (Figure 1) [12]. RO membranes do not have definable pores whereby the solution-diffusion-model can describe the transport of water and salt in such membranes [18]. RO can be grouped into seawater RO (SWRO) and brackish water RO (BWRO), according to the characteristics of the specific membranes. SWRO is usually used for a salinity close to seawater. SWRO membranes are characterized by a high salt rejection but a low permeate flux. In contrast, BWRO membranes are usually applied for feed water with a salinity range of 0.5–10 g/L and offer a higher permeate flux, a lower salt rejection, and require a lower transmembrane pressure [12]. Referring to Voutchkov [19], the average volume-related specific energy consumption ( $SEC_{V}$ ) for BWRO ranges between 0.3–2.8 kWh/m^3^ and for SWRO between 2.5–4.0 kWh/m^3^.

Nanofiltration is another cost-effective desalination technique characterized by a looser membrane compared to RO membranes, resulting in a higher permeate flux and a lower rejection of monovalent ions [18]. According to Dach [20], and Schäfer and Richards [21], the $SEC_{V}$ for SGW desalination by NF ranges between 0.2–3.5 kWh/m^3^ depending on the feed and target water concentration.

Electrochemical-driven membrane technologies such as electrodialysis and capacitive deionisation (CDI) are alternative flexible desalination technologies for brackish water with a low requirement for chemical additives [22]. Electrodialysis (reversal) has been applied in practice for several decades and is a proven energy efficient technology for brackish water desalination [23]. In contrast, CDI is an innovative technology and is seldomly used at the pilot-scale. However, CDI has become one of the most investigated electrochemical-driven desalination technologies in the last decade [24,25,26,27,28]. CDI is a cyclic desalination technique which removes charged ions from water using direct current power to generate an electric field between a positively and a negatively charged electrode which adsorb anions and cations, respectively, of saline water to generate a desalinated water stream (diluate) [29,30]. After the adsorption cycle, the rejected ions desorb into a wasted feed stream (brine) by short-circuiting the electrodes or by reversing the electrical current. The latter makes it possible to recover the electrical energy stored in the electrodes [31,32]. In order to increase the salt rejection in CDI, ion exchange membranes or ion exchange coating can be used to reduce the effect of co-ion adsorption—the adsorption of ions to an electrode carrying the same charge—and allow the electric charge to reverse during the desorption cycle [31,33]. This enhanced desalination technique is called membrane capacitive deionisation (MCDI) (Figure 1). However uncharged compounds, such as organics or biological species, are not removed by (M)CDI [14]. According to Qin et al. [34] and Zhao et al. [35], the average $SEC_{V}$ for MCDI ranges between 0.1–3.5 kWh/m^3^, treating brackish water with a salinity of 1–5 g/L and a water-recovery of 50%.

The steadily increasing demand for freshwater in regions affected by saltwater intrusion is driving the need for energy efficient desalination technologies. The desalination of SGW is—due to the relatively low salinity and low required energy consumption compared to seawater desalination—a vital solution for solving problems related to water stress and scarcity [14,36]. Many previous studies have compared the performance of RO and MCDI but have only used NaCl as the feed solution or have not fulfilled the requirements for a fair comparison of both technologies with the same boundary conditions regarding a similar level of water recovery and salt rejection [34,35,36,37].

Hence, the objective of this study is to compare the desalination performance of SGW by pressure-driven membrane desalination technologies—using NF, BWRO, and SWRO—and an electrochemical technique—using MCDI—to answer the research question: which technique has the lowest SEC regarding different desalination scenarios? Therefore, the desalination performance in this study is evaluated by the volume-related specific energy consumption ( $SEC_{V}$ ), removed ion-related specific energy consumption ( $SEC_{Ion})$ , energy efficiency, specific salt rejection, and the water recovery. Thereby, this study is not focused on the total desalination of SGW, but on partial desalination, producing “fit-for-purpose” water [27]. This was done by a small-scale experiment with constant conditions using different realistic mixed ion concentrations in the feedwater and different realistic target concentrations for the produced desalinated water. Thereby, this study evaluates the desalination performance, according to the rejection of TDS, ${\mathrm{Na}}^{+}$ , ${\mathrm{Cl}}^{-}$ , ${\mathrm{NO}}_{3}^{-}$ , and ${\mathrm{SO}}_{4}^{2-}$ . Additionally, the small-scale results for pressure-driven membrane desalination techniques were compared to simulations by using commercial membrane manufacturing software. Finally, the findings are discussed regarding future research demands.

## 2. Materials and Methods

### 2.1. Investigated Desalination Scenarios

The performance of SGW desalination was studied in small-scale experiments using synthetic SGW with three different water compositions, as shown in Table 1: a slightly SGW with 1.0 g TDS/L, a moderately SGW with 2.2 g TDS/L, and a highly SGW with 18.3 g TDS/L. To represent realistic desalination scenarios, SGW concentrations were chosen from wells located in Dalian in China, Salalah in Oman, and the Nile Delta in Egypt. The specific ion concentrations are shown in Table 2. Thereby, it is obvious that the concentrations of ${\mathrm{Na}}^{+}$ , ${\mathrm{Cl}}^{-}$ , bicarbonate ( ${\mathrm{HCO}}_{3}^{-}$ ), and ${\mathrm{SO}}_{4}^{2-}$ are lower in SGW compared to seawater. The concentrations of calcium ( ${\mathrm{Ca}}^{2+}$ ), magnesium ( ${\mathrm{Mg}}^{2+}$ ), and ${\mathrm{SO}}_{4}^{2-}$ are variable in SGW according to the hydro-geochemical reactions and biological processes in the aquifer [38,39]. The synthetic SGW was prepared by adding ${\mathrm{CaCl}}_{2}$ , KCl , ${\mathrm{MgCl}}_{2}\cdot 6H_{2}O$ , ${\mathrm{MgSO}}_{4}\cdot 7H_{2}O$ , NaCl , ${\mathrm{Na}}_{2}{\mathrm{SO}}_{4}$ , ${\mathrm{NaHCO}}_{3}$ , ${\mathrm{NaNO}}_{3}$ , and $\mathrm{Ca}{\left({\mathrm{NO}}_{3}\right)}_{2}\cdot 4H_{2}O$ into deionized water in 30–100 L basins, which were tempered according to the realistic local groundwater temperatures (±0.5 °C) in Table 2.

The target concentrations in this study were chosen according to three possible applications for partial desalinated SGW in the above-mentioned regions. Since the injection of desalinated SGW can be a used for managed aquifer recharge to artificially alter the hydraulic gradient in order to reduce or even reverse the process of saltwater intrusion [40,41], the first target concentration was set to be close to the local fresh groundwater quality [42]. The second and third target concentrations were chosen according to the recommended target concentrations for irrigation [13] and drinking water [43,44]. Hence, the different desalination scenarios were defined by three different SGW qualities and three different target concentrations (Table 2).

The sodium adsorption ratio (SAR) is defined by the ratio of ${\mathrm{Na}}^{+}$ to ${\mathrm{Ca}}^{2+}$ and ${\mathrm{Mg}}^{2+}$ . A high SAR may lead to a lower water infiltration rate into the soil, especially for water with low electric conductivity [13]. Since the rejection of divalent ions is higher than for ${\mathrm{Na}}^{+}$ for pressure-driven membrane desalination [18] as well as for MCDI [53], the SAR cannot only be managed by desalination but also by adding ${\mathrm{Ca}}^{2+}$ and ${\mathrm{Mg}}^{2+}$ in the post treatment. Since this study is focused only on desalination itself, this parameter is not considered in this study.

### 2.2. Specific Energy Consumption and Water Recovery

The mass concentrations of SGW ( $\beta_{SGW,j})$ and the target concentrations in the desalinated water ( $c_{Product,j}$ ) are shown in Table 2 for specific ions ( j ). These concentrations define the required system-scale salt rejection ( $R_{System,j}$ ; Equation (1)) and the required removed ion concentration ( $c_{i,removed}$ ; Equation (2)) which are equally defined for each desalination technique. Thereby, the molar concentration ( $c_{j})$ is calculated by the ion specific molar mass ( $M_{j})$ and the mass concentration ( $\beta_{j})$ in Equation (3).

(1) $$R_{System,j}=1-\frac{c_{Product,j}}{c_{SGW,j}}$$

(2) $$c_{j,removed}=c_{SGW,j}-c_{Product,j}$$

(3) $$c_{j}=\frac{\beta_{j}}{M_{j}}$$

The volume-related specific energy consumption ( $SEC_{V}$ ; Equation (4)) and the ion-related specific energy consumption ( $SEC_{Ion}$ ; Equation (5)), which is required to ensure the needed salt rejection in the product water, is calculated by the consumed energy ( E ) per m^3^ desalinated product water ( $V_{Product}$ ) and the removed ion concentration [34].

(4) $$SEC_{V}=\frac{E}{V_{Product}}$$

(5) $$SEC_{Ion}=\frac{E}{V_{Product}\cdot c_{j,removed}}$$

Since the desalination performances were only evaluated for mixed salt solutions and not for single salt solutions, the $SEC_{Ion}$ is therefore determined for a mixed salt solution to comply with the target concentrations for either ${\mathrm{Na}}^{+}$ , ${\mathrm{Cl}}^{-}$ , ${\mathrm{NO}}_{3}^{-}$ or ${\mathrm{SO}}_{4}^{2-}$ . The $SEC_{V}$ was thereby determined to comply with all the target concentrations.

The concentration of the synthetic SGW and the target concentration in the product water were default values in this study. The water recovery, however, can vary depending on the operation and system parameters of the used desalination technology. Additionally, a distinction was made between the module-scale water recovery ( $\gamma_{Module,w})$ and the system-scale water recovery ( $\gamma_{System,w}$ ; Equation (6)). The module-scale water recovery is the ratio of the permeate flow rate ( $Q_{DW}$ ) to the feed flow rate ( $Q_{Feed}$ ), whereas the system-scale water recovery is defined by the ratio of the product water flow rate ( $Q_{Product,w}$ ) to the SGW flow rate ( $Q_{SGW,w}$ ), which is characterised by the module-scale salt rejection ( $R_{Module,j}$ ; Equation (6)) (Figure 1).

(6) $$\gamma_{System,w}=\frac{Q_{Product,w}}{Q_{SGW,w}}$$

The module-scale salt rejection depends on the properties of the specific pressure-driven membrane desalination and MCDI modules, as well as on the specific process parameters such as pressure, applied electrical current, and flow rate. Therefore, the module-scale salt rejection ( $R_{Module,j}$ ; Equation (7)) is not equal for each desalination configuration.

(7) $$R_{Module,j}=1-\frac{c_{DW,j}}{c_{Feed,j}}$$

In case $R_{Module,j}$ > $R_{System,j}$ , the partial desalinated flow rate ( $Q_{DW,w}$ ) was blended by using an adequate bypass flow rate ( $Q_{Bypass,\;w}$ ) of SGW (Figure 1) to achieve a higher system-scale water recovery for pressure-driven membrane desalination due to the high salt retention of the membranes [12,19].

The theoretical minimum volume-related specific energy consumption ( $SEC_{min,V}$ ) for separating a saline solution into a partial desalinated stream and a concentrated brine can be calculated by the second law of thermodynamics [15,54]. The $SEC_{min,V}$ is thereby independent of the desalination technique and used in this paper to calculate the energy efficiency by the ratio of $SEC_{min,V}$ to $SEC_{V}$ of the respective desalination processes. Therefore, it is assumed that $SEC_{min,V}$ for separating, equals the minimum Gibbs free energy for salt-water mixtures, neglecting the energy loss by friction or heat. Since two ideal solutions—a high concentrated solution (here: brine) and a low saline solution (here: partial desalinated water)—which are initially separated by an impermeable membrane, react spontaneously to a mixed solution (by removing the impermeability of the membrane) due to diffusion, the change in the Gibbs free energy $\;(∆G_{mix}$ ) can be calculated by the increase in entropy of the mixed solution ( $∆S_{mix};$ Equation (7)) [55].

(8) $$∆G_{mix}=-T\cdot ∆S_{mix}=R_{g}T\sum x_{j}\ln x_{j}$$

where *R_g_ is* the ideal gas constant ( $8.314\;\frac{kg\;m^{2}}{s^{2}mol\;K}$ ), *T* the temperature ( K ), and $x_{j}$ the mole fraction of the considered salt. Equation (8) can be transcribed to the following formula (Equation (9)) to calculate the $SEC_{min,V}$ for desalination [56].

(9) $$SEC_{min,V}=∆G_{mix}\approx iR_{g}T\left[\frac{c_{Feed}}{\gamma }ln\left(\frac{c_{Brine}}{c_{Feed}}\right)-c_{Product}\;ln\left(\frac{c_{Brine}}{c_{Product}}\right)\right]$$

where i is the specific van’t Hoff factor. Since this study is focused on the mixed saltwater compositions, $SEC_{min,V}$ was calculated for the mixed salt concentrations in Table 2.

### 2.3. Pressure-Driven Membrane Desalination

#### 2.3.1. Specific Energy Consumption

The energy-consuming factor for pressure-driven membrane desalination is the feed pressure. The feed pressure needs to be adjusted to ensure the required permeate (and concentrate) flow rate, considering the friction losses and the concentration polarization in a membrane module. Therefore, the feed pressure in a small-scale design differs from that in a big-scale design. In order to calculate the SEC for a realistic large-scale desalination plant, the production flow rate was set to 25 m^3^/h, which represents a small-desalination plant [19]. The required feed pressure, depends on the properties of the membrane and the properties of the solutions on each side of the membrane and inside the membrane [57]. In this study, the required feed pressure, which should be representative of a realistic plant, was calculated according to the solution–diffusion model [18]. The required feed pressure emerges from the water and salt permeability, which can be identified by small-scale experiments, a literature review, or by the usage of the commercial design software of the membrane manufactures (WAVE Design Software 1.58 by the DOW Chemical Company, Midland, MI, USA [58] and LewaPlus^®^ Design Software 2.1.1 by LANXESS Deutschland GmbH, Cologne, Germany [59,60]). In this study, the water and salt permeability were obtained by small-scale experiments.

The water permeability ( A , Equation (10)) is calculated by the permeate flow rate ( $Q_{DW}$ ), the membrane surface area ( S ) and the net driving pressure [12,18].

(10) $$A=\frac{Q_{DW}}{S\cdot \left(∆P-∆\pi \right)}$$

The net driving pressure results from the pressure difference ( ∆P ) regarding the feed pressure, the permeate pressure and the pressure drop, and the osmotic pressure difference between the feed side and the permeate side of the membrane (∆π ).

The salt permeability ( B ; Equation (11)) is modelled as the Fickian diffusion by the specific salt flux ( $N_{S}$ ) and the difference of the average concentrate-side concentration ( $\beta_{fc}$ ) and the permeate concentration ( $\beta_{DW}$ ) of the small-scale experiments [12,18].

(11) $$B=\frac{N_{S}}{\left(\beta_{fc}-\beta_{DW}\right)},$$

The ideal desalination membrane has a high water permeability paired with a near-zero salt permeability for ions which need to be rejected [61]. Since both water and salt permeability vary with the operating temperature, $A_{T}$ - and $B_{T}$ -coefficients were calculated according to temperatures in Table 2, using the simplified design equations of LANXESS Deutschland GmbH (Köln, Germany) [60,62] and FilmTec™ (DuPont de Nemours, Wilmington, NC, USA) [63]. In order to compare the experimental determined water permeability with the literature, $A_{25{}^{\circ}C}$ coefficients were normalised to 25 °C.

The required feed pressure for each desalination scenario was set according to Equation (10). Therefore, a single-module design with 6-elements in series was selected. The number of parallel modules was adapted to the active surface area of the specific membrane modules, the required permeate flow, and the module-scale recovery. The module-scale recovery was set to 60% for NF and BWRO, and 40% for SWRO, according to the design guidelines for the specific membranes [58,59,62,63].

In total, the SEC for pressure-driven membrane desalination depends on the water and salt permeability of the membranes, the water temperature, and the salt concentration in the feedwater. The SEC for pressure-driven membrane desalination ( $SEC_{PDMD,V})\;$ was calculated considering the feed flow ( $Q_{Feed})$ , the feed pressure ( $P_{Feed})$ and the concentrate pressure ( $P_{Concentrate})$ , the water recovery, and the efficiency of the feed pump and energy recovery device (Equation (12)).

(12) $$SEC_{PDMD,V}=\frac{Q_{Feed}\left(P_{Feed}-P_{Concentrate}\left(1-\gamma_{Module,w}\right)\;\eta_{ERD}\right)}{\eta_{Pump}Q_{Product}},$$

In actual desalination plants, energy recovery devices are used in order to reduce the SEC by recovering the remaining pressure of the brine into mechanical energy via either turbines or pressure exchangers [14]. Here, the efficiency of the pump ( $\eta_{Pump}$ ) and the efficiency of the energy recovery device ( $\eta_{ERD}$ ) were assumed to be both 80% [64,65].

#### 2.3.2. Experimental Procedure

The small-scale pressure-driven membrane desalination experiments were performed with one single element using three different dense flat sheet membranes with a high NaCl -retention:

- DOW FILMTEC™ Flat Sheet NF90;
- Lewabrane^®^ RO B085 HF;
- DOW FILMTEC™ SW30XLE.

The desalination performance was investigated at the Leibniz Institute of Polymer Research Dresden using an OSMO Inspector 2 (Convergence Industry B.V., Enschede, Netherlands) and a stainless steel membrane holder of SIMA-tec^®^ GmbH, Schwalmtal, Germany (UF10-85). The active membrane area was set to 210 mm × 40 mm. Since the height of the feed channel was set to 1.016 mm by the membrane holder, the same diamond shaped spacer with a height of 40 mil was used for all experiments. All experiments were performed using a constant feed pressure at two different pressure stages, which were adjusted to the recommended permeate flux ranges of the membrane manufacturers [58,59,63]. Since the lowest possible constant feed flow was 65 kg/h, the crossflow velocity was set to 0.44 m/s for all pressure-driven membrane desalination tests.

Before each desalination test, the membranes were wetted and compacted gradually with deionized water until the permeate flux was stable. Since the retention for highly SGW needed to be higher than 95%, NF was not evaluated for desalination of highly SGW. All desalination tests were performed in triplicate.

The permeate flow and the transmembrane pressure were measured online in each test period for 1–2 h. The concentration of single ions, the pH, and the electric conductivity were measured in the feed, concentrate, and permeate sample for each experimental run. The qualitative sample analyses were carried out at the Institute of Urban and Industrial Water Management at the Technische Universität Dresden. The pH and conductivity were measured using a HQ40D portable multi meter from Hach, Düsseldorf, Germany, with a PHC 301 electrode and a CDC 401 electrode. The cations were measured by atomic absorption spectroscopy using a SpectrAA 220FS Varian, Agilent, Palo Alto, CA, USA, according to DIN 38406-3:2002-03 [66] and DIN 38406-13/14:1992-07 [67,68]. The anions were measured by ion chromatography using an ICS 3000 from DIONEX, Sunnyvale, CA, USA, according to DIN EN ISO 10304-1:2009-07 [69], whereby the ${\mathrm{HCO}}_{3}^{-}$ were determined by the acid capacity using 888 Titrando, Metrohm, Herisau, Switzerland according to DIN 38409-7:2005-12 [70].

### 2.4. Membrane Capacitive Deionisation

#### 2.4.1. Specific Energy Consumption

The most energy-consuming factor of MCDI is the electric energy needed to generate an electric field between the electrodes. The energy demand of the feed pressure can be neglected, since the feedwater is just flowing by and not pressed through a membrane [34,35,71]. In this study, the specific energy consumption for MCDI ( $SEC_{MCDI,V}$ ; Equation (13)) is calculated by integrating the electrical current ( $I_{el,ads}$ ) and the cell voltage ( $V_{cell}$ ) over the adsorption time during three complete cycles in steady state. Since the electrical currents were reversed during desorption until the voltage dropped to 0 V, a part of the consumed energy during adsorption was recovered during desorption [32].

(13) $$SEC_{MCDI,V}=\;\frac{\int_{0}^{t_{ads}}(V_{cell,ads}I_{el,ads})dt-\int_{ads}^{t_{cycle}}(V_{cell,des}I_{el,des})dt}{V_{P}},$$

The $SEC_{V}$ (Equation (4)) and $SEC_{Ion}$ (Equation (5)) were calculated as for the pressure-driven membrane desalination for a production of 25 m^3^/h desalinated water using Python 3.7 (Python Software Foundation, Wilmington, DE, USA).

The electrosorption process in CDI can be described by the formation of the electrical double layers within the micropores of the carbon electrodes [72]. In the past, the desalination performance was predicted mainly by the Gouy–Chapman–Stern model [73,74,75], considering the presence of a diffuse layer and an inner dense (Helmholtz/Stern) layer between the diffuse and the electrode surface; by the modified-Donnan model [76,77,78,79], considering the overlapping of the diffuse layer within the micropores and an ion specific chemical attraction term; or by the amphoteric-Donnan model [80], considering fixed basic and acidic chemical charges in the micropores. Including the ion transport, which can be described by the Nernst–Planck equation, these models can be used to predict the energy consumption and the ion rejection [78,79]. In rare studies in which the electrosorption of mixed salt were modelled, the selectivity of specific ions with the same valence was included by an experimentally determined attraction or affinity term [81]. In total, the ion specific adsorption and the interaction between ions are time dependent with regard to the ion specific valence, diffusion coefficient, hydrated radius, the applied electricity, the pH, and the ion concentration [53,82,83,84]. The ion volume exclusion interactions resulting in a preferred adsorption of ions with a smaller hydrated radius, were described by Suss and Guyes et al. [85,86]. However, as currently shown in the study of Tsai et al. [83], the influence of ion exchange membranes can reduce the affinity and thereby the selectivity. According to the knowledge of the authors, a multi-ion transport model does not exist, including the selective interactions in the micropore and the exclusion effects by the ion exchange membranes. Therefore, the rejection and energy consumption for MCDI were calculated in this study by experimental results.

#### 2.4.2. Experimental Procedure

The MCDI small-scale experiments were conducted with a C 03 25 DDRG MCDI module integrated in the CapDi Pilot Unit from 2010 of Voltea B.V., Sassenheim, Netherlands, at the Technische Universität Dresden. The pilot plant was controlled by using the “Demo Unit software” from Voltea B.V. (2010) and Tera Term v. 4.92. The total electrode surface area of the module was 3.7 m^2^. In this study, we wanted to achieve a constant diluate quality. Therefore, the desalination tests were performed using a constant electrical current [87]. The duration during charging of the electrodes were defined by the adoption time until the maximum voltage was reached. To obtain a low SEC, the electrical current was reversed during the desorption until the cell voltage ( $V_{cell}$ ) dropped back to 0 V [32].

The required electrical current ( $I_{el}$ ) to obtain the wanted ion rejection, was calculated by the Faraday’s constant (F); the aimed average concentration of the diluate ( $c_{D}$ ), and the current efficiency ( $\eta_{I})$ , which was assumed to be approximately 80% (Equation (14)) [31,32,77].

(14) $$I_{el}=\frac{F\cdot Q_{F}\cdot \sum \left(c_{SGW,i}-c_{D}\right)}{\eta_{I}},$$

According to Equation (14), a higher electrical current was selected in order to achieve the higher required salt retention. However, the MCDI module used in this study is not designed for desalination of highly SGW. Hence, desalination tests were performed only with slightly SGW and moderately SGW. In this study, 10 A, 14 A, 18 A, 22 A and 26 A were applied for desalination of slightly SGW, and 20 A, 40 A, 45 A, 50 A and 55 A for moderately SGW. The feed flow ( $Q_{F}$ ) was constant and set to 1.0 L/min. The maximum voltage ( $V_{cell,max}$ ) was set at 1.2 V with the exception of runs at 55 A where $\;V_{cell,max}$ was set to 1.6 V since the module-scale water recovery was too low for a $V_{cell,max}$ of 1.2 V. The variable module-scale water recovery for the MCDI ( $\gamma_{MCDI,\;Module}$ ) depends on the electrical current selected, the feed water quality and the feed flow and was calculated by the duration of adsorption and desorption and the pre-purifying duration (Equation (15)).

(15) $$\gamma_{MCDI,\;Module}=\frac{∆t_{adsorption}-∆t_{pre-purifying\;}}{∆t_{adsorption}+∆t_{desorption}},$$

where $∆t_{\mathrm{adsorption}}$ is the duration of adsorption and $∆t_{\mathrm{desorption}}\;$ is the duration of desorption. The pre-purifying duration ( $∆t_{\mathrm{pre}-\mathrm{purifying}\;})\;$ is the duration which was needed to reach ≥90% of the potential salt rejection to circumvent the contamination of the diluate with salts remaining from the desorption mode.

After three complete adsorption and desorption cycles, the desalination performance was defined to be in steady state. The single ions were analyzed in feed water samples and diluate samples in mixed samples of three complete cycles in steady state. Here, the same analytical methods as for the pressure-driven membrane desalination processes were used. In addition, the conductivity of the inlet and outlet flow of the MCDI module, and the cell voltage were measured online.

## 3. Results

### 3.1. Minimum Specific Energy Consumption

**Figure 2:**
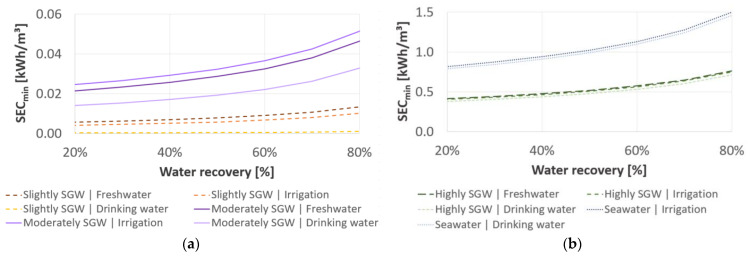
Minimum volume-related specific energy consumption ( $SEC_{min,V}$ ) as a function of water recovery: (**a**) for slightly SGW and moderately SGW; (**b**) for highly SGW and seawater regarding the target concentrations for fresh groundwater, water for irrigation and drinking water.

Independent of desalination technology, the $SEC_{min,V}$ rises with an increasing feed concentration, salt rejection and an increasing water recovery (Equation (9)). However, the $SEC_{min,\;V}$ varies with the required salt retention according to the target concentration in the partial desalinated water (Table 2). As shown in Figure 2, the $SEC_{min,\;V}\;$ is 1.1 kWh/m^3^ for seawater desalination at a water recovery of 60%, whereby the $SEC_{min,\;V}\;$ is ≤0.01 kWh/m^3^, ≤0.04 kWh/m^3^ and ≤0.6 kWh/m^3^ for slightly SGW, moderately SGW and highly SGW, respectively. Therefore, the $SEC_{min,\;V}$ is higher for the target concentrations regarding the local freshwater and irrigation guidelines compared to the target concentrations for drinking water.

### 3.2. Desalination Performance of Pressure-Driven Membrane Desalination

**Figure 3:**
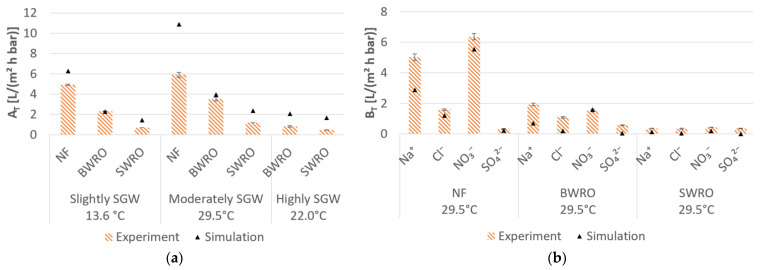
Average permeability: (**a**) water permeability ( $A_{T}$ ), (**b**) salt permeability ( $B_{T}$ ) for desalination of moderately SGW at 29.5 °C. The bars show the 95% confidence interval for the experimental results. NF: nanofiltration; BWRO: brackish water reverse osmosis; SWRO: seawater reverse osmosis.

Both the water and salt permeability follow the order of NF > BWRO > SWRO (Figure 3). The experimentally determined water permeability varied between 0.46 L/(m^2^ h bar) and 5.91 L/(m^2^ h bar) (Figure 3a). The highest water permeability was obtained for desalination of moderately SGW due to a feedwater temperature of 29.5 °C, despite the feedwater concentration in moderately SGW being higher than that in slightly SGW. The highest variances for the water permeability were obtained for NF and SWRO, for desalination of moderately SGW and highly SGW, respectively. The highest salt permeability was observed for ${\mathrm{Na}}^{+}$ and ${\mathrm{NO}}_{3}^{-}$ (Figure 3b).

The experimental determined normalized water permeability at 25.0 °C follows the order of slightly SGW > moderately SGW > highly SGW (Table 3). In general, the water and salt permeability obtained by the software was higher compared to experimental results (Figure 3, Table 3). With the exception of highly SGW, the experimentally obtained water permeabilities generally corresponds well with the literature (Table 3). Moreover, the feedwater concentration had a higher impact on the water permeability in the experiments compared to the modelled results (Table 3).

**Figure 4:**
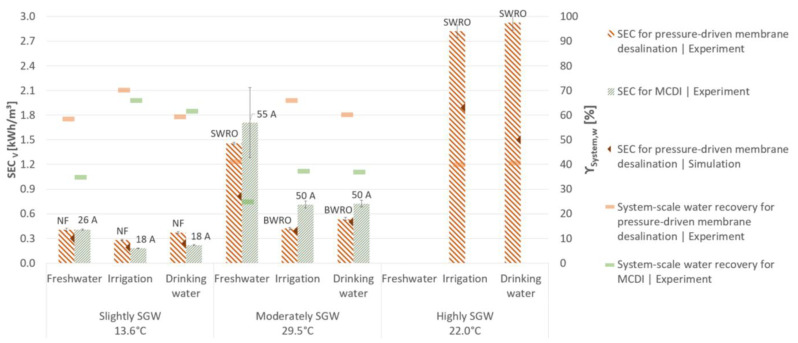
Volume-related specific energy consumption ( $SEC_{V}$ ) and system-scale water recovery ( $\gamma_{System,w}$ ). The $SEC_{V}$ is shown for the most efficient desalination configuration of pressure-driven membrane desalination and membrane capacitive deionisation (MCDI) (the respective type of applied membrane and applied electrical current is given above the bars). Empty bars represents configurations not suitable for the aimed target concentration. The error bars represent the 95% confidence interval.

**Figure 5:**
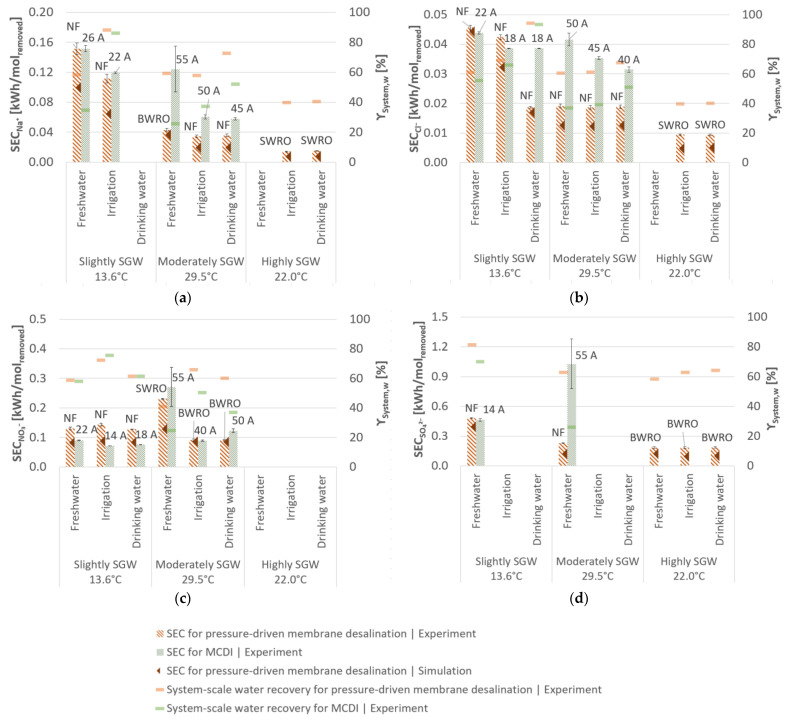
Removed ion-related specific energy consumption ( $SEC_{Ion}$ ) and system-scale water recovery ( $\gamma_{System,w}$ ) for (**a**) ${\mathrm{Na}}^{+}$ , (**b**) ${\mathrm{Cl}}^{-}$ , (**c**) ${\mathrm{NO}}_{3}^{-}$ , (**d**) ${\mathrm{SO}}_{4}^{2-}$ . The $SEC_{Ion}$ is shown for the most efficient desalination configuration of pressure-driven membrane desalination and MCDI (the respective type of applied membrane and applied electrical current is given above the bars). Empty bars represent configurations not suitable for the aimed target concentration (highly SGW) or that no salt retention was required (slightly SGW, moderately SGW). The error bars represent the 95% confidence interval.

The lowest SEC was achieved with a high water permeability, a low salt permeability, and low a required salt rejection (Figure 3, Figure 4 and Figure 5). The modelled SEC was lower than the experimental SEC (Figure 4 and Figure 5) which can be explained by the higher water permeability and lower salt permeability determined by the simulation software (Figure 3). Hence, the average experimentally determined $SEC_{PDMD,V}$ varies depending on the feed salt concentration and target salt concentration. For desalination of slightly SGW with NF, the SEC ranged from 0.3 to 0.4 kWh/m^3^, whereas this was 0.4 to 1.5 kWh/m^3^ for desalination of moderately SGW with BWRO or SWRO (Figure 4). For desalination of highly SGW with SWRO, the SEC ranged from 2.8 to 2.9 kWh/m^3^. The achieved system-scale water recovery in one-stage design ranged between 48–70, 41–66, and 40% for NF, BWRO, and SWRO, respectively.

The highest energy efficiency ( $SEC_{min,\;V}$ / $SEC_{V})$ was achieved with <18% for highly SGW. The energy efficiency for slightly SGW and moderately SGW was ≤5 and ≤10%, respectively. The normalization of the water and salt permeability to 25 °C would change the $SEC_{PDMD,V}$ for slightly SGW, moderately SGW, and highly SGW by −25, +10 and −7%, respectively.

According to the high permeability for ${\mathrm{Na}}^{+}$ and ${\mathrm{NO}}_{3}^{-}$ for pressure-driven membrane desalination (Figure 3) and the low required salt rejection for ${\mathrm{SO}}_{4}^{2-}$ (Table 2), the highest experimentally determined $SEC_{PDMD,Ion}$ values were achieved for ${\mathrm{Na}}^{+}$ with ≤0.15 kWh/mol_removed_, for ${\mathrm{NO}}_{3}^{-}$ with ≤0.23 kWh/mol_removed_, and for ${\mathrm{SO}}_{4}^{2-}$ with ≤0.48 kWh/mol_removed_ (Figure 5). For ${\mathrm{Cl}}^{-}$ the $SEC_{PDMD,Cl^{-}}$ ranged between 0.01–0.05 kWh/mol_removed_. Since the $SEC_{Ion}$ depends on the required salt rejection, this parameter is not intended for comparing SEC of different desalination scenarios for pressure-driven membrane desalination, but rather for the comparison of the same scenarios for pressure-driven membrane desalination and MCDI. In general, the pH in the permeate was lower (6.1–7.3) than in the feed (8.1–8.3).

### 3.3. Desalination Performance of Membrane Capacitive Deionisation

**Figure 6:**
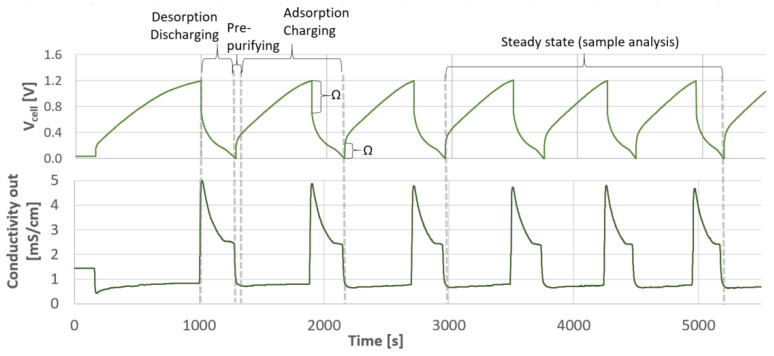
Cell voltage ( $V_{cell}$ ) and conductivity of the outflow of the MCDI module during desalination of slightly SGW at 14 A (Ω: ohmic resistance).

In contrast to pressure-driven membrane desalination, the module-scale water recovery was not a default parameter for MCDI. The water recovery decreases with increasing the applied electrical current (Table 4), due to the decreasing relation of the produced diluate volume to the produced concentrate volume caused by the higher ohmic resistance in the solution (Figure 6) [35,94,95].

The SEC during adsorption—as well as the recovered energy during desorption—increases at higher electrical currents (Table 4). Since the energy recovery increase is lower regarding the simultaneous increase in the required energy demand during adsorption, the total energy demand is greater at higher applied electrical currents (Figure 4). The $SEC_{MCDI,V}$ , the recovered energy, and the module-scale salt rejection are positively correlated with the applied electrical current. The module-scale water recovery is negatively correlated with the applied electrical current. The $SEC_{MCDI,V}$ in Figure 4 varies from 0.2 to 0.4 kWh/m^3^ at 18–26 A and 0.7 to 1.7 kWh/m^3^ at 50–55 A, respectively, for slightly SGW and moderately SGW. The high confidence interval for the experiments with 55 A and a maximum voltage of 1.6 V results from the higher variance of adsorbed ions at a higher salt rejection. The achieved system-scale water recovery ranged between 35 and 66% for MCDI desalination of slightly SGW, whereas this was 25 to 37% for moderately SGW (Figure 4). The energy efficiency for slightly SGW and moderately SGW was ≤8 and ≤4%, respectively.

**Figure 7:**
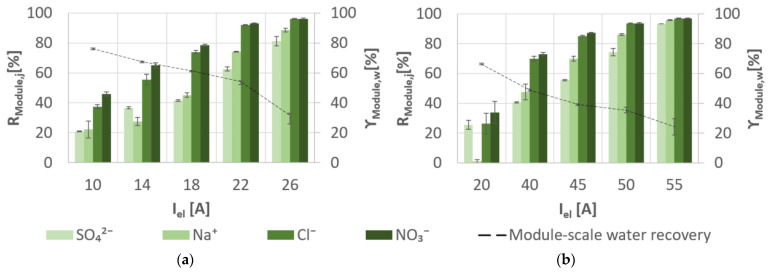
Specific module-scale salt rejection ( $R_{Module,j})$ and module-scale water recovery ( $\gamma_{Module,w})$ for experimental desalination: (**a**) slightly SGW and (**b**) moderately SGW using MCDI.

The ion selectivity in Figure 7 follows, in general, the order of ${\mathrm{NO}}_{3}^{-}$ > ${\mathrm{Cl}}^{-}$ > ${\mathrm{Na}}^{+}$ > ${\mathrm{SO}}_{4}^{2-}$ . Additionally, the module-scale salt rejection varied between 1–96% and increased with increasing the applied electrical current. The lowest $SEC_{MCDI,Ion}$ values were obtained for ${\mathrm{NO}}_{3}^{-}$ and ${\mathrm{Cl}}^{-}$ with 0.07–0.27 kWh/mol_removed_ and 0.03–0.04 kWh/mol_removed_, respectively (Figure 5). The $SEC_{MCDI,Ion}$ for ${\mathrm{Na}}^{+}$ and ${\mathrm{SO}}_{4}^{2-}$ ranged between 0.06–0.15 kWh/mol_removed_ and 0.46–1.03 kWh/mol_removed_, respectively. The pH in the diluate was slightly lower (6.6–8.3) than in the feed (7.3–8.7).

## 4. Discussion

Independent of the method used, the lowest $SEC_{V}$ values were generally achieved for desalination of slightly SGW due to the lower required salt rejection (Figure 2 and Figure 4). Therefore, the experimentally determined $SEC_{V}$ for desalination of slightly SGW was up to 36% lower for MCDI than for pressure-driven membrane desalination, regarding the target concentrations for irrigation and drinking water showing a similar system-scale water recovery. Even though the average $SEC_{Ion}$ for ${\mathrm{Na}}^{+}$ (Figure 5) was marginal higher for MCDI for these desalination scenarios, here, MCDI overall showed a better desalination performance due to the high salt rejection for ${\mathrm{NO}}_{3}^{-}$ and ${\mathrm{Cl}}^{-}$ , even at low applied electrical currents (Figure 7). As a result, MCDI can prove to be an energy efficient desalination technology especially for ${\mathrm{NO}}_{3}^{-}$ -rich groundwater. However, the ion selectivity of mixed ion solutions within MCDI cannot yet be fully mathematically described. Therefore, more research is needed to understand and intensify these selective effects if necessary.

If a higher salt rejection for desalination of slightly SGW is required to comply with the target concentrations of local freshwater, pressure-driven membrane desalination technologies are more advantageous due to their higher system-scale water recovery, even though MCDI showed a similar $SEC_{V}$ (Figure 4). According to the desalination scenarios of moderately SGW and highly SGW, pressure-driven membrane desalination technologies show a lower $SEC_{V}$ and a higher system-scale water recovery and are therefore more suitable than MCDI. Thereby, the appropriate membrane (NF, BWRO or SWRO) has to be chosen according to the specific salt retention, system design and further boundary conditions of the desalination scenario.

The laboratory tests were examined under ideal conditions. Even though precipitation was not detected in the concentrate during short-term experiments, the solubility limit was exceeded by simulating big-scale pressure-driven membrane desalination. Other substances, which were not examined in this study, such as silica, iron or organic matter, can further enhance the scaling and fouling potential [12,96] and will therefore affect the SEC and water recovery. Hereby, scaling and fouling effects have been thoroughly investigated for pressure-driven membrane desalination and can be minimized by the operation procedure and scaling inhibitors. Since in MCDI the rejection is not driven by pressure, clogging effects are assumed to be lower for MCDI compared to pressure-driven membrane desalination [26]. The influence of scaling and fouling on the desalination performance of MCDI is, however, controversially discussed in the literature. Therefore, more research, including the temperature effect on scaling and fouling in realistic long-term and large-scale experiments, is mandatory in this study field [14]. Since SGW can further contain methane and hydrogen sulphide due to decomposition of organic matter under anoxic or anaerobic conditions [97], the pretreatment needs to be adjusted regarding the specific water composition and the used desalination technology.

The obtained $SEC_{V}$ for MCDI in this study corresponds well with results of Qin et al. [34] and Zhao et al. [35]. The experimental and simulated achieved $SEC_{V}$ by pressure-driven membrane desalination are in accordance with values generally reported in the literature [19,20,21]. Despite the water and salt permeability determined in NF and BWRO desalination experiments differing from simulated results, the experimentally obtained permeability results are still in accordance with the literature (Table 3). In total, the simulated permeability results generally underestimate SEC compared to the experimental results (Figure 4 and Figure 5). The experimental determined water permeability for highly SGW desalination was lower compared to the literature. Deviations in permeability obtained by experiments and simulations might result from different flow characteristics in small-scale experiments, usual production variabilities of the membranes and concentration polarisation. However, the influence of the saline concentration on the water permeability [98] for slightly SGW and moderately SGW desalination experiments could not be determined by the design software and was only visible by the experimental results (Table 3). Consequently, the simulated as well as the experimental determined SEC for pressure-driven membrane desalination should be considered for the performance comparison with MCDI.

In this study, the desalination performance was examined for one-stage configurations to ensure similar conditions for pressure-driven membrane desalination and MCDI. According to Werber et al. [99] and Shrivastava et al. [100], the application of multiple stages or concentrate recirculation can achieve lower SEC values with higher water recoveries. The SEC and the water recovery for MCDI can be increased by reducing the pre-purifying duration and by reducing—or even stopping—the flow during the desorption mode [37,101]. However, a higher water recovery results in a higher scaling potential and higher concentrations in the rejected brine, which might not comply with corresponding guidelines for surface water discharge [102].

Overall, the experimental and simulated results in this study showed higher $SEC_{V}$ (Figure 4) than $SEC_{min,V}$ values (Figure 2) due to irreversible energy losses. Therefore, the energy efficiency for pressure-driven membrane desalination increased with higher salt rejection (slightly SGW < moderately SGW < highly SGW) due to lower $SEC_{Ion}$ for these desalination scenarios. In contrast, the energy efficiency decreased for MCDI with higher salt rejections. Typical losses for pressure-driven membrane desalination are defined by inefficiencies of feed pump and energy recovery devices (ERD), the membrane and module performance, and losses caused by the system design [15,100]. According to Werber et al. [61] future research should be focused on a higher salt selectivity for pressure-driven membrane desalination. Conversely, typical losses for MCDI include the ionic resistive and electric resistive losses as well as the parasitic losses from Faradaic charge-transfer reactions [103]. In order to operate the MCDI close to the thermodynamic limit, further research is needed to reduce the resistive and parasitic energy losses caused by an increase in the specific salt rejection [33,103,104].

For pressure-driven membrane desalination as well, as for MCDI, the feedwater temperature is an important factor influencing the energy efficiency, the SEC, and the water recovery. In this study, the desalination performance was evaluated for the specific temperature of the respective SGW. The $SEC_{V}$ for pressure-driven membrane desalination decreases with a rising feedwater temperature due to the higher impact of the increasing water permeability compared to the increasing salt permeability. Since the resistance of an aquareous solution decreases with its temperature, the SEC should decrease, as is the case for electrodialysis [105]. However, according to the experimental CDI study by Mossad and Zou [106], the salt rejection was inversely related to the feedwater temperature probably due to a lower adsorption capacity, the tendency of metal ions to escape from the electrode surface or hydrophobic to hydrophilic transitions on the surface of the activated carbon. In total, a higher feedwater temperature would result in higher SEC for MCDI and lower SEC for pressure-driven membrane desalination.

The module-scale water recovery and the module-scale salt rejection of NF, BWRO, and SWRO were linked to narrow operation guidelines of the module specifications for the flow rate of the feed, permeate, and concentrate. The total flow rates therefore depend on the specific design of the membrane elements. The adjustment of the flow rate for MCDI was, however, flexibly adjustable according to the required salt rejection and the applied electrical current. Therefore, MCDI shows great potential for meeting flexible desalination demands in terms of salt rejection and water recovery [26]. Due to the flexibility by easily adapting the flow or the electric energy demand, MCDI is as electrodialysis compatible with the unstable and oscillating energy supply associated with renewable energy resources, such as photovoltaic or wind energy [23].

**Figure 8:**
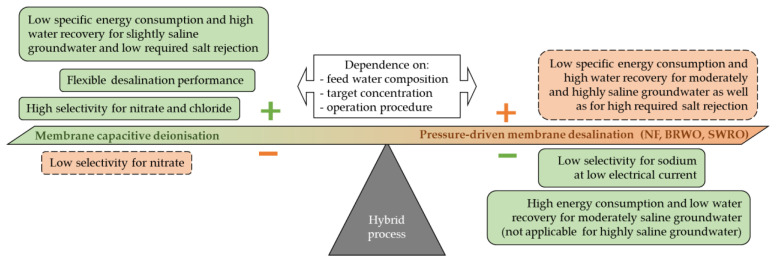
Benefits and drawbacks of pressure-driven membrane desalination (red and dashed frame) and membrane capacitive deionisation (green and solid frame) detected in this study.

This study analysed the SEC for pressure-driven membrane desalination technologies and MCDI to compare the desalination performance for mixed salt solutions. In order to compare the total costs, further cost parameters, such as the capital costs, the required area for the desalination plant, and the adapted pre- and posttreatment costs need to be considered as well. Moreover, both desalination technologies—pressure-driven membrane desalination and MCDI—can benefit from each other in hybrid processes (Figure 8). Due to the adjustable selectivity effects of MCDI, this technique could be used in hybrid configurations as a pretreatment or second-permeate stage to increase the energy efficiency of pressure-driven membrane desalination technologies [107,108].

## 5. Conclusions

In this study, the desalination performance was examined for three different realistic SGW concentrations using NF, BWRO, SWRO, and MCDI. Our experimental results indicate that pressure-driven membrane desalination and MCDI show—depending on the operation procedure—different selectivity towards ${\mathrm{Na}}^{+}$ , ${\mathrm{Cl}}^{-}$ , ${\mathrm{NO}}_{3}^{-}$ , and ${\mathrm{SO}}_{4}^{2-}$ . The desalination performance of the specific desalination technology should, therefore, not only be evaluated for different feed concentrations, but also for mixed ion concentrations regarding different target concentrations.

The lowest SEC values for a low level of required salt rejection, such as for slightly SGW (TDS = 1 g/L), were achieved with MCDI and NF. Thereby, the lowest SEC values were obtained with MCDI for slightly SGW with regard to the guideline concentrations for irrigation and drinking water. However, if a higher salt rejection is required, as for the target concentration for local freshwater, NF demonstrated a better desalination performance than MCDI, due to a higher water recovery. Pressure-driven membrane desalination—such as BWRO and SWRO—demonstrated, independent of the target concentrations under the respective boundary conditions, a better desalination performance for TDS concentrations ≥2 g/L.

Even though the experiments of this study confirm that pressure-driven membrane desalination technologies show a higher energy efficiency regarding higher saline concentrations, we showed that MCDI is particularly suitable for desalination of ${\mathrm{NO}}_{3}^{-}$ -rich groundwater, as well as for flexible boundary conditions. However, more research is needed to evaluate the impact of mixed ion solutions in long-term MCDI studies.

## Figures and Tables

**Table 1:** Classification of water salinity based on the mass concentration of total dissolved solids (TDS) and chloride $\left({\mathrm{Cl}}^{-}\right)$ [10,11].

Classification of Water Salinity	TDS	$Cl^{-}$
	mg/L	mg/L
Freshwater	0–500	<100
Slightly saline groundwater	500–1500	100–250
Moderately saline groundwater	1500–7000	250–500
Highly saline groundwater	7000–35,000	500–10,000
Seawater	>35,000	>10,000

**Table 2:** Ion mass concentration (β) (mg/L), conductivity (µS/cm) and temperature (°C) of seawater, slightly SGW, moderately SGW, highly SGW and the target concentrations (local fresh groundwater quality, global guideline concentrations for irrigation water for sensitive crops and global guideline concentrations for drinking water quality).

	Seawater	Dalian, China		Salalah, Oman		Nile Delta, Egypt		Global	
		Freshwater	Slightly SGW	Freshwater	Moderately SGW	Freshwater	Highly SGW	Irrigation	Drinking Water
**Ref.**	**[45,46]**	**[47]** **w. n. 3**	**[47]** **w. n. 13**	**[48]** **w. n. 29**	**[49]** **w. n. 4**	**[50]** **w. n. 19**	**[50]** **w. n. 10**	**[13]**	**[43,44]**
${\mathrm{Ca}}^{2+}$	412	65 ^SAR^	172	102 ^SAR^	223	40 ^SAR^	546	n.s. ^SAR^	n.s.
$Na^{+}$	**10,782**	**19 ^SAR^**	**81**	**38 ^SAR^**	**340**	**20 ^SAR^**	**4658**	**69 ^SAR^**	**200**
${\mathrm{Mg}}^{2+}$	1284	12 ^SAR^	32	16 ^SAR^	107	14 ^SAR^	1130	n.s. ^SAR^	n.s.
$K^{+}$	399	3	2	1	7	9	100	n.s.	n.s.
$Cl^{-}$	**19,353**	**34**	**273**	**90**	**702**	**30**	**10,645**	**106**	**250**
${\mathrm{HCO}}_{3}^{-}$	113	126	118	234	242	160	154	92	n.s.
$SO_{4}^{2-}$	**2712**	**66**	**97**	**27**	**176**	**19**	**1057**	**200**	**250**
$NO_{3}^{-}$	**n.s.**	**47**	**230**	**24**	**416**	**n.s.**	**n.s.**	**130 ^1^**	**50**
TDS	35,055	372	1004	532	2213	292	18,289	450	600
Conductivity	n.s.	560	1740	794	4510	440	32,000	700	n.s.
pH	8	7.8	7.6	n.s.	7.1	7.8	7.2	6.5–8.5	
Temperature ^2^	n.s.	13.1	13.6	29.5 ^2^	29.5 ^2^	22.0 ^2^	22.0 ^2^	n.s.	n.s.

n.s. = not specified, w. n. = well number, ^SAR^ = sodium adsorption ratio, ^1^ nitrate-sensitive crops might have a lower target concentration. ^2^ groundwater temperature, which were not given in the respective literature, was assumed to be the mean air temperature above the land surface [51,52]. Bold: specific ions, which exceed the target concentrations. SGW: Saline groundwater.

**Table 3:** Average water permeability at 25 °C ( $A_{25{}^{\circ}C}$ ).

Membrane	$A_{25\;{}^{\circ}C}\;\mathrm{in}\;L/(m^{2}\;h\;\mathrm{bar})$						
	Experiments			Simulation			Literature
	Slightly SGW	Moderately SGW	Highly SGW	Slightly SGW	Moderately SGW	Highly SGW	
DOW FILMTEC™ NF90-400/34i	7.37	5.18	-	9.43	9.55	-	4.0–10.2 [88,89,90]
LEWABRANE^®^ RO B085 HF 4040	3.45	3.09	0.91	3.43	3.47	2.29	2.0–5.3 ^1^ [61,91,92]
DOW FILMTEC™ SW30XLE-400i	1.03	1.04	0.51	2.17	2.07	1.87	0.9–3.0 ^1^ [6,91,93]

^1^ Since no water permeability coefficients for the specific membranes were found, results for similar polyamide thin-film composite membranes are shown.

**Table 4:** Average values of energy balance, module-scale water recovery ( $\gamma_{Module,w})$ and module-scale salt rejection ( $R_{Module,TDS})$ regarding the electrical current ( $I_{el}$ ) for MCDI desalination experiments (without blending).

Figure	$I_{el}$	$\gamma_{Module,w}$	$SEC_{MCDI,V}\;\mathrm{during}\;\mathrm{Charging}$	Recovered Energy during Discharging	Energy Recovery	$R_{Module,TDS}$
	A	%	kWh/m^3^	kWh/m^3^	%	%
Slightly SGW	10	76.11	0.15	0.01	9.57	37.10
	14	67.48	0.20	0.03	14.12	54.41
	18	61.46	0.27	0.05	18.01	68.09
	22	54.29	0.39	0.08	20.89	87.18
	26	31.45	0.62	0.15	23.98	93.49
Moderately SGW	20	66.34	0.29	0.06	20.95	25.18
	40	48.87	0.68	0.24	36.04	64.64
	45	39.10	0.94	0.34	36.59	80.27
	50	35.42	1.12	0.35	31.29	90.46
	55	24.22	2.44	0.69	28.05	96.31

## Data Availability

The data presented in this study are available on request from the corresponding author.
