# Correction: Rosentreter et al. Partial Desalination of Saline Groundwater: Comparison of Nanofiltration, Reverse Osmosis and Membrane Capacitive Deionisation. *Membranes* 2021, *11*, 126

**DOI:** 10.3390/membranes16010036

**Published:** 2026-01-06

**Authors:** Hanna Rosentreter, Marc Walther, André Lerch

**Affiliations:** 1 Chair of Process Engineering in Hydro Systems, Institute of Urban and Industrial Water Management, Faculty of Environmental Sciences, Technische Universität Dresden, 01062 Dresden, Germany; 2 Chair of Forest Biometrics and Forest Systems Analysis, Department of Forest Sciences, Faculty of Environmental Sciences, Technische Universität Dresden, 01062 Dresden, Germany

There was an error in the original publication. The well number of referenced freshwater quality shown for the Nile Delta in Table 2 [1] was “19” and not “10” from reference [50]. Since the water quality data were correctly shown in Table 2 from well number 19, it had no influence on the correctness of the results.

In Equation (7), there was a typing error in the presentation of the equation. The correct equation is $R_{Module,j}=1-\frac{c_{DW,j}}{c_{Feed,j}}$.

The results of the paper are based on the correct equation. The authors state that the scientific conclusions are unaffected. This correction was approved by the Academic Editor. The original publication has also been updated.

## References

[1] Rosentreter H., Walther M., Lerch A. (2021). Partial Desalination of Saline Groundwater: Comparison of Nanofiltration, Reverse Osmosis and Membrane Capacitive Deionisation. Membranes.

